# Large-Scale Synthesis of Hierarchical Porous MOF Particles via a Gelation Process for High Areal Capacitance Supercapacitors

**DOI:** 10.3390/nano13101691

**Published:** 2023-05-21

**Authors:** Yujie Sun, Fei Shi, Bo Wang, Naien Shi, Zhen Ding, Linghai Xie, Jiadong Jiang, Min Han

**Affiliations:** 1 State Key Laboratory of Organic Electronics and Information Displays, Institute of Advanced Materials, Nanjing University of Posts & Telecommunications, Nanjing 210023, China; 2 Strait Laboratory of Flexible Electronics (SLoFE), Strait Institute of Flexible Electronics (SIFE, Future Technologies), Fujian Normal University, Fuzhou 350117, China

**Keywords:** metal–organic framework, particles, gelation, hierarchical porous, supercapacitor

## Abstract

Metal–organic frameworks (MOFs) with hierarchical porous structures have been attracting intense interest currently due to their promising applications in catalysis, energy storage, drug delivery, and photocatalysis. Current fabrication methods usually employ template-assisted synthesis or thermal annealing at high temperatures. However, large-scale production of hierarchical porous metal–organic framework (MOF) particles with a simple procedure and mild condition is still a challenge, which hampers their application. To address this issue, we proposed a gelation-based production method and achieved hierarchical porous zeolitic imidazolate framework-67 (called HP-ZIF67-G thereafter) particles conveniently. This method is based on a metal–organic gelation process through a mechanically stimulated wet chemical reaction of metal ions and ligands. The interior of the gel system is composed of small nano and submicron ZIF-67 particles as well as the employed solvent. The relatively large pore size of the graded pore channels spontaneously formed during the growth process is conducive to the increased transfer rate of substances within the particles. It is proposed that the Brownian motion amplitude of the solute is greatly reduced in the gel state, which leads to porous defects inside the nanoparticles. Furthermore, HP-ZIF67-G nanoparticles interwoven with polyaniline (PANI) exhibited an exceptional electrochemical charge storage performance with an areal capacitance of 2500 mF cm^−2^, surpassing those of many MOF materials. This stimulates new studies on MOF-based gel systems to obtain hierarchical porous metal–organic frameworks which should benefit further applications in a wide spectrum of fields ranging from fundamental research to industrial applications.

## 1. Introduction

Metal–organic frameworks (MOFs), as a typical type of hybrid porous materials, have attracted considerable attentions since their discovery and have long been being a research hotspot in materials science [1,2,3]. The distinct large specific surface area of MOFs can provide a substantial number of active sites for energy storage [4,5,6], gas adsorption [7,8,9], catalysis [10,11], and filtration [12,13]. However, most typical MOF materials are composed of microporous structures with pore size smaller than 2 nm [14,15], which limit the adsorption or permeation of large molecules, thereby hindering the transport of substances in MOF materials. This limitation restricts the advantages of MOF porous structures in energy storage, heterogeneous catalysis, drug delivery, and other fields. Currently, chemists and materials scientists are striving to introduce mesoporous structures (2–50 nm) and even macroporous structures (>50 nm) into MOFs to obtain hierarchical microporous–mesoporous–macroporous structures. The synthesis of hierarchical porous MOF structures involves several methods, including ligand extension, topological design, post-modification strategies (e.g., acid etching and thermal treatment), and template synthesis strategies (e.g., soft and hard template methods). However, each of these methods present various limitations. The ligand extension method can be assembled by designing longer ligand chains to form mesopores, but this method requires complex organic synthetic procedures [16,17]. The method of topological design is limited by specific molecules and it requires theoretical modeling assistance [18,19]. The thermal annealing method removes a portion of the groups from the molecular chain of the material by a thermal reaction in order to create micropores in nanomaterials. However, it is not widely applicable due to its difficult operation and high energy costs [20,21,22]. As for template methods, such as employing polymer beads or surfactants, they are limited in further applications due to the complex post-treatment procedures and the consequent constricted yield [23,24]. Therefore, the current methods for synthesizing porous MOF materials encounter the problems of long synthetic procedures, high energy consumption, complex operations, and limited yield. It is still a challenge to prepare hierarchical porous MOF particles with simple procedures, mild conditions, and high yields.

In this communication, we report a novel approach on synthesizing hierarchical porous metal–organic framework (MOF) particles with small size and hierarchical porous structures by utilizing a supramolecular metal–organic gelation process. The approach involves employing a local zeolite imidazole skeleton, zeolitic imidazolate framework-67 (Co(MIM)_2_; MIM: 2-methylimidazole), as the building block to form the gel. The internal structure of the metal–organic gel is primarily maintained by intermolecular forces, which are weaker than the covalent bonds present in ordinary polymeric gels. This characteristic imparts remarkable mechanical stimulus responses to the gel, allowing it to undergo rapid sol–gel transitions under cyclic stress loading. Furthermore, this property facilitates coating process of the gel, enabling the production of extremely thin film. Additionally, due to the high concentration of raw materials employed in the reaction, the method allows for large-scale production of ZIF-67 porous nanoparticles in a small volume. The slow diffusion rate of solutes inside the low molecular weight gel (LMWG) also promotes the generation of a large number of defects during nanoparticle growth, resulting in a high specific surface area and improved electrochemical properties for the nanoparticles. Consequently, we were able to synthesize HP-ZIF67-G nanoparticles with a large number of mesopores on a large scale. Solid-state symmetric supercapacitor was fabricated using HP-ZIF67-G as the active electrode material, and it exhibits a high capacitance of 2500 mF cm^−2^, which obviously surpasses the non-porous orthododecahedral ZIF-67. This work should benefit the design of new hierarchical porous MOFs and stimulate further applications in the fields of energy storage, catalysis, or chemical sensors.

## 2. Materials and Methods

### 2.1. Materials

2-methylimidazole (2-MIM), trimethylamine and Polyvinyl alcohol (PVA) were purchased from Shanghai Aladdin Biochemical Technology Co., Ltd. (Shanghai, China), and Co(NO_3_)_2_·6H_2_O, KCl, KOH, H_2_SO_4_, ethanol (EtOH), hydrochloric acid (HCl), polyvinylidene fluoride (PVDF), and isopropyl alcohol were purchased from Sinopharm Chemical Reagent Co., Ltd. (Shanghai, China), and deionized water was obtained from a EPED-40TJ ultra-pure water system (CAYN lnc., Shanghai, China). All reagents and solvents were used as received without further purification.

### 2.2. Synthesis of ZIF-67 LMWG

Co(NO_3_)_2_·6H_2_O (0.524 g, 1.8 mmol) and 2-methylimidazole (0.591 g, 7.2 mmol) were dissolved in 1.2 mL EtOH in a 10 mL vial, respectively. Then, trimethylamine (1.1 mL, 30 wt%, aqueous solution) was added to the 2-methylimidazole solution, and it was sonicated for 5 min until the residual solid was completely dissolved. A Co(NO_3_)_2_ solution was slowly added dropwise to the 2-MIM solution, and a large amount of purple solid precipitation was observed at the bottom of the glass bottle. After adding all the Co(NO_3_)_2_ solution, the glass bottle was vigorously shaken until the gel was uniform. Finally, the obtained gel was subjected to sonication in a sonicator for 30 min and left it stand at room temperature for 12 h.

### 2.3. Preparation of Porous HP-ZIF67-G Nanoparticles

The gel was placed in a vacuum freeze dryer to completely remove the solvent and obtained a purple-white ZIF-67 nanoparticle powder. The powder was dispersed in mixed deionized water and ethanol. The suspension was separated by centrifugation at 800 r/min for 5 min, and the upper layer of purple liquid was discarded. Then the precipitate was repeatedly washed with deionized water and ethanol, respectively, until the upper layer of liquid became colorless and transparent after centrifugation. Finally, the obtained purple solid was washed three times using ethanol to wash away the deionized water and then placed into a vacuum drying oven at 75 °C for 12 h to obtain clean hierarchical porous ZIF-67 nanoparticles (HP-ZIF67-G).

### 2.4. Preparation of the HP-ZIF67-G/CC Electrode

The working electrodes were prepared by mixing the as-prepared composite, super-conductive carbon black, and polyvinylidene fluoride with the mass ratio of 80:15:5. The mixture was ground adequately to form a slurry, then coated onto carbon cloth (CC, 1.0 cm × 1.0 cm) and dried in a vacuum oven at 75 °C for 24 h.

### 2.5. Preparation of the HP-ZIF67-G/PANI/CC Electrode

The electro-deposition of polyaniline (PANI) and the subsequent electrochemical tests were performed using a Shanghai Chen Hua Instrument CHI660e. PANI was electro-deposited by the previously reported cyclic voltammetry method with ZIF-67 as the working electrode, and the desired electrode was obtained by cycling 75 times between −0.2 V and 1.0 V potentials at a scanning rate of 10 mV s^−1^ in a mixed solution of KCl (3 mol L^−1^) and aniline (0.1 mol L^−1^). The Ag/AgCl electrode was used as the reference electrode. Then, the obtained HP-ZIF67-G/PANI/CC electrode was washed with deionized water and dried in a vertical vacuum oven at 75 °C for at least 12 h. About 300 mg of HP-ZIF67-G nanoparticles was obtained.

### 2.6. Preparation of the PVA-H_2_SO_4_ Gel Electrolyte

An amount of 15 mL of sulfuric acid solution (1 mol L^−1^) was poured into a beaker, placed into an oil bath, and the temperature was raised to 85 °C. Then, 1.5 g PVA was added into the beaker and stirred until the solution was completely clear and transparent. The solution was cooled to room temperature and left to stand for 24 h before being used.

### 2.7. Preparation of ZIF-67 Crystals

ZIF-67 crystals were synthesized by a similar method reported previously in the literature [23]. An amount of 10 mL of deionized water was first put into a bottle, and 1 mL of Co(NO_3_)_2_·6H_2_O (0.193 mmol, 0.056 g) aqueous solution was added and stirred to form a uniform solution. An amount of 1 mL 2-MIM aqueous solution (2.73 mmol, 0.224 g) was slowly added and the mixture was continuously stirred for 20 s. Then, the mixed solution was left to stand at room temperature for 24 h. A purple product was produced at the bottom of the glass bottle, and this product was collected by centrifugation. It was washed and centrifuged with fresh methanol three times. Finally, the product was dried under vacuum at 75 °C for 12 h.

### 2.8. Instruments

Field emission scanning electron microscopy (SEM) images were taken on a Hitachi S-4800 field emission scanning electron microscope. Transmission electron microscopy (TEM) images were obtained on a Hitachi HT-7700 transmission electron microscope. X-ray powder diffractometer (XRD) images were taken on Bruker D8 ADVANCE X-ray diffractometer using Cu Kα radiation (1.54056 Å). X-ray photoelectron spectroscopy (XPS) images were taken on an Axis Supra X-ray photoelectron spectrometer. N_2_ adsorption–desorption tests were executed on a Micromeritics ASAP 2050 analyzer. Cyclic voltammetry (CV), chronopotentiometry (GCD), and impedance were taken on a CHI660e electrochemical workstation. The experiments were carried out in a three-electrode system with a working electrode, a platinum plate counter electrode, and an Ag/AgCl reference electrode. The electrolyte was 1.0 mol L^−1^ KCl aqueous solution. For the dual-electrode solid-state device system, PVA-H_2_SO_4_ gel electrolyte was employed.

## 3. Results and Discussion

In this study, high concentrations of Co(NO_3_)_2_ and 2-methylimidazole aqueous solution with a small amount of trimethylamine (TMA) were reacted to obtain ZIF-67 LMWG. ZIF-67 LMWG was freeze-dried and characterized using SEM and TEM images, revealing the formation of numerous small-sized porous ZIF-67 nanoparticles. By coating HP-ZIF67-G on carbon cloth and electrodepositing PANI, we obtained a porous composite electrode material with an excellent capacitive performance.

### 3.1. Formation of ZIF-67 LMWG

**Figure 1:**
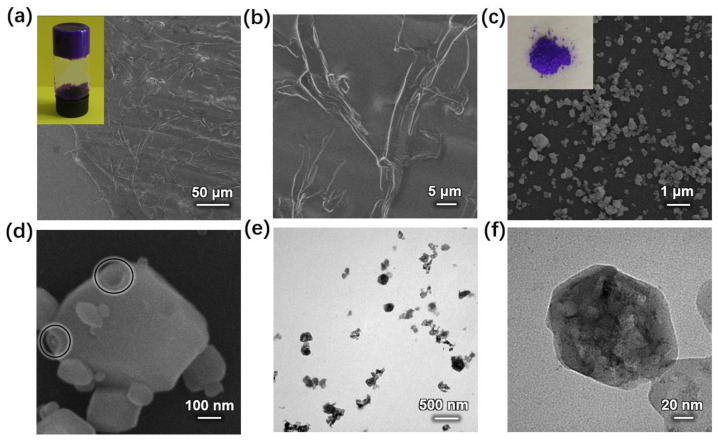
(**a**,**b**) SEM images of gel film coated on a silicon wafer (inset: the photograph of ZIF-67 LMWG); (**c**,**d**) low and high magnification SEM images of HP-ZIF67-G particles (inset: the photograph of HP-ZIF67-G); (**e**,**f**) corresponding TEM images of HP-ZIF67-G particles.

The ZIF-67 LMWG synthesized under the conditions in Table S1 (Table S1, No. 1) are shown in Figure 1a. As observed, the formed gel exhibited a purple color. Furthermore, it exhibited a remarkable mechanical stability, as it did not collapse or deform when inverted. In addition, the gel exhibited thixotropic properties, allowing it to change from the gel to sol state via the application of shear force and return to the gel state within 3 min when it was left to stand. This unique gel enables the material to display exceptional ductility, which can be harnessed to produce ZIF-67 films. Figure 1a,b shows the formed film of ZIF-67 LMWG. By coating ZIF-67 LMWG on silicon wafers, a uniform ZIF-67 film can be obtained. The film can completely cover the silicon wafer and the surface is smooth and flat.

A controlled experiment on the tuning of the reaction conditions is shown in Figure S1 and Table S1. When the concentration of 2-methylimidazole was halved (Table S1, No. 2), the gel tended to collapse. Similarly, when the concentration of cobalt ions was further halved (Table S1, No. 3), it could not form a stable gel. Thus, a decrease in cobalt ion concentration led to a complete collapse of the gel. In addition, further increasing the concentrations of cobalt ions and 2-methylimidazole by 1.5 times (Table S1, No. 5) also failed to produce the desired gel, which is probably due to the products exceeding the solvent capacitance of the formed particles and consequently impeding the formation of the gel net structure. Moreover, changing the solvent from ethanol to an equal amount of deionized water (Table S1, No. 6) led to a turbid liquid product that could not form a gel. By adjusting the reactant concentration, the product can eventually become a ZIF-67 gel with a lower fluidity. It should be noted that ZIF-67 gel are obtained at reactant concentrations much higher than other methods for the synthesis of ZIF-67 [25,26,27], thus resulting in a substantially higher volumetric yield of the product.

### 3.2. Characterization of HP-ZIF67-G Particles

**Figure 2:**
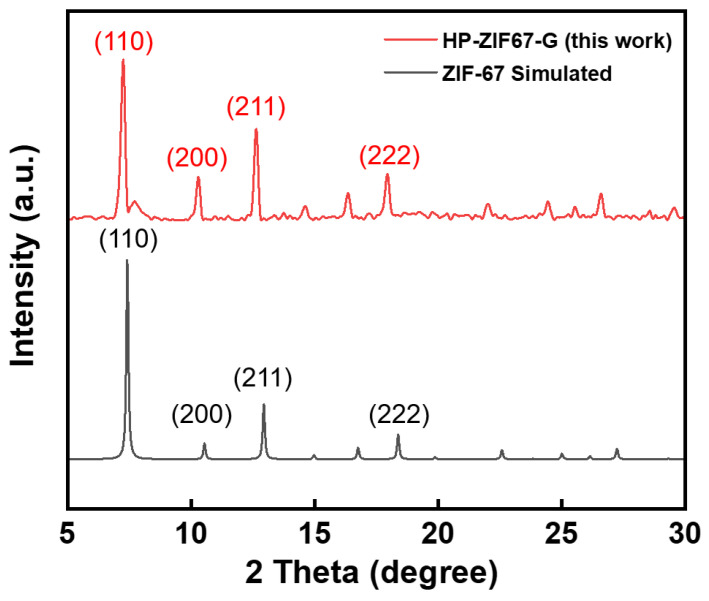
XRD pattern of HP-ZIF67-G nanoparticles (red) and the simulated XRD pattern of ZIF-67 (black).

Figure 1c shows SEM images of the obtained HP-ZIF67-G nanoparticles from the ZIF-67 gel. It is revealed that the diameter of nanoparticles was in the range of 200–500 nm. In the enlarged SEM image (Figure 1d), the pores can be clearly seen on the surface of the HP-ZIF67-G nanoparticles. Figure 1e,f shows the corresponding TEM images of the HP-ZIF67-G nanoparticles. As can be clearly seen, there are a lot of mesopores inside the particles. The hierarchical porous structure denotes nanoparticles with a large specific surface area which could be beneficial to their electrochemical properties. Figure 2 illustrates the X-ray diffraction (XRD) pattern of the ZIF-67 nanoparticles prepared by high concentration reaction. It exhibits several sharp peaks that match the simulated XRD pattern of ZIF-67 (CCDC number: 671074) [15]. Some residual peaks in the XRD pattern of the HP-ZIF67-G nanoparticle can be seen, which may be due to phase impurities arising from lattice dislocation caused by the coordination of trimethylamine and cobalt ions. However, the XRD spectra exhibit a clear consistency between the primary diffraction peaks of HP-ZIF67-G and the simulated XRD patterns of ZIF-67, indicating the material’s robust crystalline structure despite the extensive distribution of hierarchical pores within the sample. 

**Figure 3:**
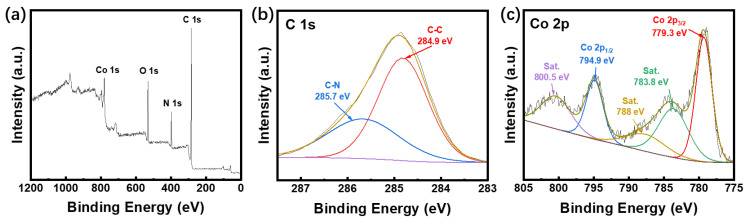
(**a**) XPS survey spectrum of HP-ZIF67-G; (**b**) corresponding core-level fine XPS spectra for C 1s, (**c**) corresponding core-level fine XPS spectra for Co 2p.

To investigate the surface element species and their corresponding chemical states, X-ray photoelectron spectroscopy (XPS) was applied (Figure 3a–c). The full XPS survey spectrum of HP-ZIF67-G (Figure 3a) indicated the co-existence of C, N, and Co from HP-ZIF67-G and O from air. The high-resolution XPS C 1s spectra in Figure 3b show two peaks, corresponding to C-C (284.9 eV) and C-N (285.7 eV). As for Co 2p, the high-resolution XPS plots in Figure 3c can be divided into two peaks of Co 2p_1/2_ (794.9 eV) and Co 2p_3/2_ (779.3 eV) and three satellite peaks at 800.5 eV, 788 eV, and 783.8 eV, respectively [28,29], indicating the Co(II) in HP-ZIF67-G.

**Figure 4:**
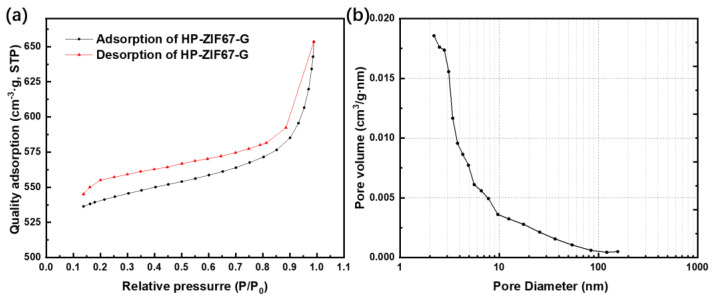
(**a**) N_2_ adsorption–desorption isotherm curves of HP-ZIF67-G; (**b**) corresponding pore–size distribution of HP-ZIF67-G.

To obtain an insight into the specific surface area and pore structures of typical HP-ZIF67-G, N_2_ adsorption–desorption measurements were further recorded. As shown in Figure 4a, the sample exhibits the combined characteristics of type III isothermal curves. The corresponding pore–size distribution plots (Figure 4b) can provide further evidence for this. Calculated from the adsorption branch using the Barrett–Joyner–Halenda method, the specific surface area of HP-ZIF67-G is about 1765 m^2^ g^−1^ and the total pore volume is 1.01 cm^3^ g^−1^, which is much higher than standard ZIF-67 nanoparticles (1500 m^2^ g^−1^ and 0.79 cm^3^ g^−1^, respectively) [30]. As seen in pore–size distribution plot, we can observe a large number of pores locating at the size range of 2–100 nm. Mesopores and macropores provide mass transport channels inside the nanoparticles, and thus improve the performance when used as capacitor materials.

### 3.3. Mechanistic Investigation

Typically, when ZIF-67 grew in a low concentration solution system (lower than 0.2 M metal ions), dodecahedron nanocrystals are observed (as seen in Figure S2). The formation of MOFs involves three stages: the nucleation stage, the growth stage, and the ripening stage. During the nucleation stage, metal ions and organic matter coordinate and this leads to the formation of small MOF crystal nuclei. If we increase the precursor concentration, the nucleation rate accelerates, resulting in smaller nuclei interspaces and greater growth competition between nuclei, which inhibits the growth of nanoparticles. This competitive growth mechanism prevents HP-ZIF67-G nanoparticles from growing into orthododecahedra crystals.

**Figure 5:**
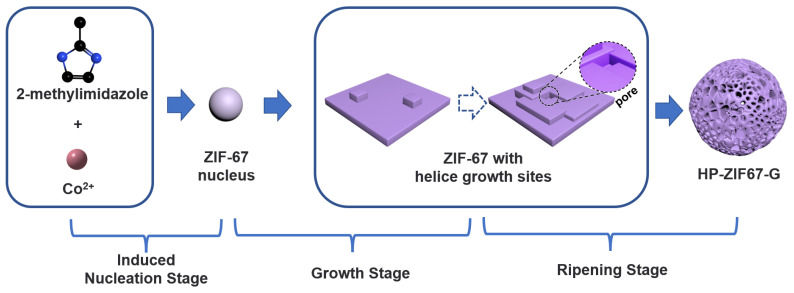
Proposed schematic of the formation process of HP-ZIF67-G.

Next, gelation is initiated during the following growth and ripening stages. In the gel system, both trimethylamine and 2-methylimidazole can undergo coordination reactions with cobalt ions, but the coordination kinetics of cobalt ions with 2-methylimidazole is stronger than that of trimethylamine. The above two coordination reactions may lead to misalignment of the lattice during the growth of ZIF-67 nanoparticles [31,32,33]. Meanwhile, ZIF-based materials tend to exhibit a helical growth approach [34,35]. As shown in Figure 5, during the growth of zeolitic imidazolate frameworks, helix growth is observed in the nuclei, which involves the formation of 2D surface nuclei on the surface and subsequent lateral spreading, then forming growth steps on the surface. Since it is difficult to perform tracking experiments on ZIF-67 nanoparticles in gels, we analyzed the growth process of ZIF-67 under the same conditions as that of HP-ZIF67-G but in the absence of trimethylamine to prove the existence of such a process. The TEM image of the products is shown in Figure S3, and we can observe that the flaky nanosheets are curled up into flower-shaped nanosheets with extrusion, suggesting an earlier lateral extension growth stage. Besides, as can be clearly seen, there is a thickness inhomogeneity in the nanosheets, which may arise from a similar helical growth process. During the following growth stage in LMWGs, due to the decreased Brownian motion of the substances and the coordination competition of trimethylamine in the gel, defects and pores are often left. Finally, in the ripening stage, HP-ZIF67-G nanoparticles engulf each other, the pores inside the nanoparticles are closed, and HP-ZIF-67 hierarchical porous nanoparticles are formed.

### 3.4. HP-ZIF67-G-Based Supercapacitors 

**Figure 6:**
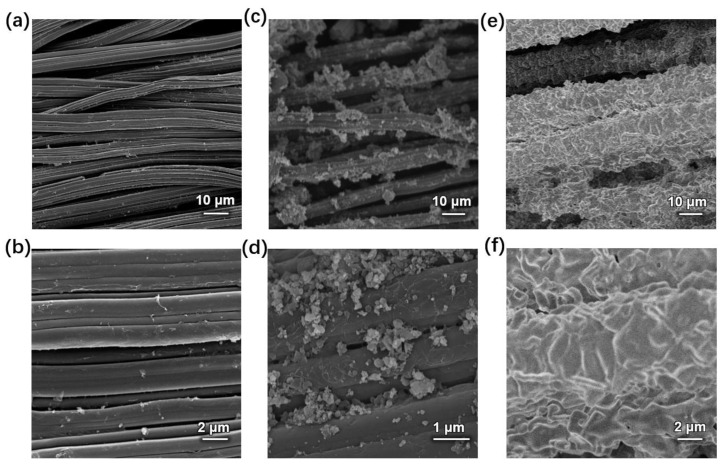
Low- and high-magnification SEM images of (**a**,**b**) blank carbon cloth, (**c**,**d**) HP-ZIF67-G on the carbon cloth, and (**e**,**f**) HP-ZIF67-G and PANI on the carbon cloth, respectively.

HP-ZIF67-G was coated on carbon cloth (mixed with carbon black and polyvinylidene fluoride). Furthermore, an electrodeposition process was used to modify a layer of the polyaniline material on the electrode. A photograph of the HP-ZIF67-G/PANI/CC electrode (Figure S4) and SEM images of the electrode are shown in Figure S4 and Figure 6, respectively. Figure 6a,b is the clean carbon cloth. After the HP-ZIF67-G nanoparticles, carbon black and polyvinylidene fluoride adhesive were coated onto the carbon cloth, and they were tightly adhered to the carbon cloth (Figure 6c,d). Then, polyaniline was electrodeposited onto the surface of HP-ZIF67-G/CC by cyclic voltammetry to form the HP-ZIF67-G/PANI/CC electrode, where the CV curve is shown in Figure S5. SEM images show that the polyaniline tightly wrapped around the surface of the carbon cloth, interconnecting the isolated ZIF-67 crystals and serving as a bridge for electron transport between the HP-ZIF67-G nanoparticles (Figure 6e,f). Energy dispersive spectroscopy (EDS) tests of HP-ZIF67-G/PANI/CC (Figure S6) showed that there is cobalt, carbon, and nitrogen in the electrode.

**Figure 7:**
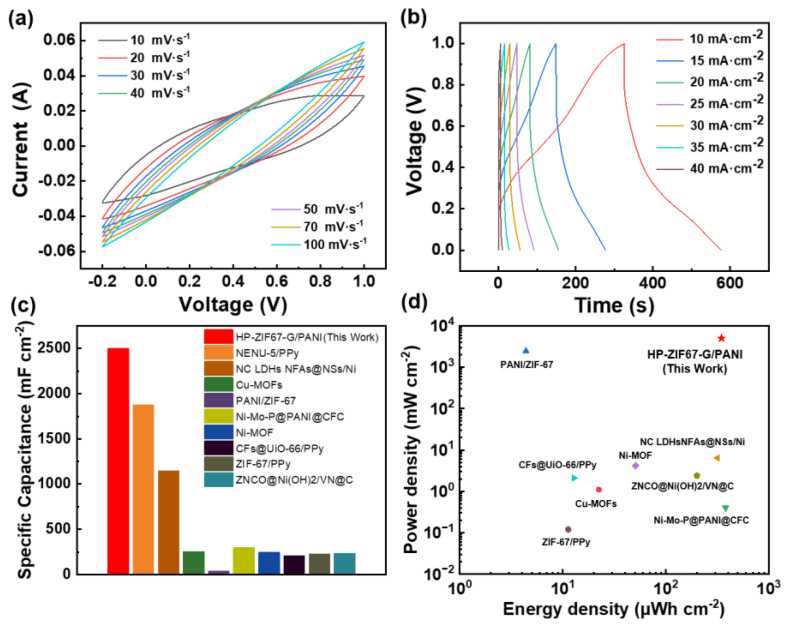
(**a**) CV curves of the HP-ZIF67-G/PANI/CC symmetric device with PVA-H_2_SO_4_ gel electrolyte; (**b**) GCD curves of the HP-ZIF67-G/PANI/CC symmetric device with PVA-H_2_SO_4_ gel electrolyte; (**c**) specific capacitance of the solid-state supercapacitor−based HP-ZIF67-G and other reported electrode materials; (**d**) Ragone plots of HP-ZIF67-G and the listed materials [36,37,38,39,40,41,42,43].

Two identical HP-ZIF67-G/PANI/CC electrodes were used as the positive and negative electrodes of the symmetric supercapacitor device, and ca. 250 mg PVA-H_2_SO_4_ gel was employed as the electrolyte. Figure S7a illustrates a digital image of the symmetric supercapacitor device. As seen, the CV curve (Figure 7a) does not exhibit a prominent redox peak and exhibits electric double layer capacitor (EDLC) characteristics at high scan rate, and as the scan rate decreases, a more pronounced bulge gradually becomes apparent, indicating the material’s pseudocapacitance characteristics. The constant current charge–discharge cycling (GCD cycle) curves have a high voltage window of 0–1V (Figure 7b), and it has a high specific capacitance of 2500 mF cm^−2^ at a current density of 10 mA cm^−2^. Figure 7c shows the performance comparison of HP-ZIF67-G/PANI electrode material and other MOF-conducting polymer-based materials. As seen, the HP-ZIF67-G/PANI electrode is at the highest level. Besides, according to the Ragone plots shown in Figure 7d, the highest energy density is 346 μWh cm^−2^ with a power density of 5000 mW cm^−2^, surpassing the values achieved for MOF-conducting polymer-based supercapacitors reported previously [36,37,38,39,40,41,42,43].

**Figure 8:**
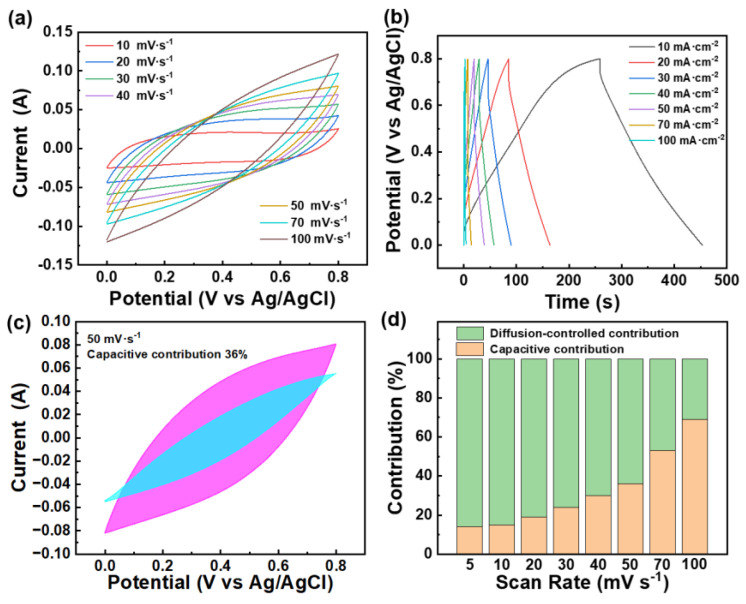
(**a**) CV curves of HP-ZIF67-G/PANI/CC in a 1.0 mol L^−1^ KCl aqueous solution electrolyte; (**b**) GCD curves of HP-ZIF67-G/PANI/CC in a 1.0 mol L^−1^ KCl aqueous solution electrolyte. (**c**) Capacitance contribution of the HP-ZIF67-G/PANI/CC electrode in KCl aqueous solution electrolyte at 50 mV s^−1^, for detailed data refer to Figure S8; (**d**) capacitance contribution ratio between surface capacitive process and the diffusion–controlled process at different scan rates.

In addition, the HP-ZIF67-G/PANI/CC-based device also exhibits remarkable performance in liquid electrolytes. We prepared a liquid-state three-electrode supercapacitor device using the HP-ZIF67-G/PANI/CC electrode as the positive electrode, a platinum sheet electrode as the counter electrode, an Ag/AgCl electrode as the reference electrode, and 3 mol L^−1^ KCl aqueous solution as the electrolyte. Digital images of the supercapacitor device are shown in Figure S7b. As seen in Figure 8a, redox peaks can be observed in the CV curves, and according to the GCD curves (Figure 8b), the capacitance can reach 2625 mF cm^−2^ at the current density of 10 mA cm^−2^. The kinetic process of the energy storage reaction was calculated according to the literature [44,45], and the results are shown in Figure 8c,d. It can be seen that the HP-ZIF67-G/PANI/CC electrode exhibits a mixed capacitance type of EDLC and pseudocapacitance at high scan rates, while it mainly exhibits pseudocapacitance characteristics at low scan rates. The EDLC capacitance is mainly provided by HP-ZIF67-G and the pseudocapacitance is mainly provided by PANI.

To further investigate the electrochemical properties of the hierarchical porous structure, we tested the performance of the HP-ZIF67-G/CC and ZIF-67 (orthododecahedral ZIF-67 crystal)/CC supercapacitor. The supercapacitor performance of HP-ZIF67-G and ZIF-67 crystals in PVA-H_2_SO_4_ gel electrolyte is shown in Figure S9. As seen, the performance of HP-ZIF67-G/CC (15 mF cm^−2^) is approximately 20% higher than ZIF-67/CC (12.5 mF cm^−2^). Moreover, we prepared ZIF-67/PANI/CC supercapacitor devices under the same conditions as HP-ZIF67-G/PANI/CC. The results are shown in Figures S10 and S11. In liquid electrolyte, the ZIF-67/PANI/CC supercapacitor device exhibited an areal capacitance of 2037 mF cm^−2^. HP-ZIF67-G/PANI/CC (2625 mF cm^−2^) improved by 29% as compared to ZIF-67/PANI/CC. In the solid-state symmetric supercapacitor device, the ZIF-67/PANI/CC exhibited an areal capacitance of 1750 mF cm^−2^ and HP-ZIF67-G/PANI/CC (2500 mF cm^−2^) exhibited an improvement of 30% compared to ZIF-67.

**Figure 9:**
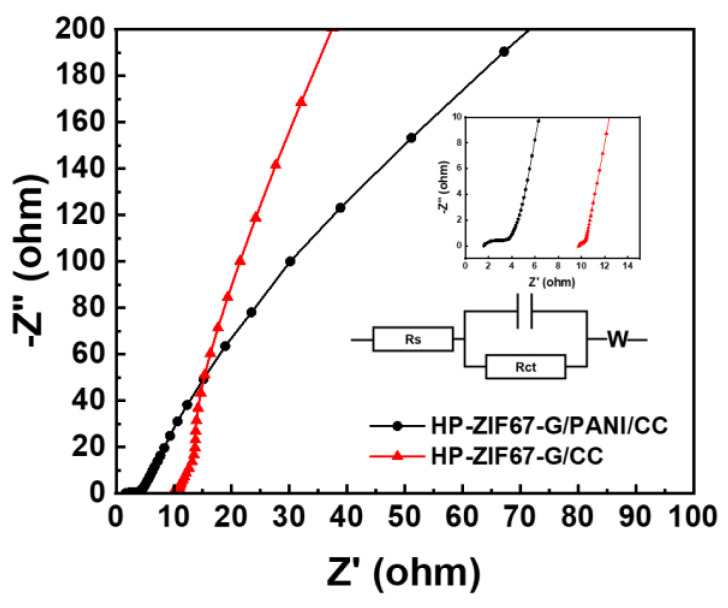
Nyquist electrochemical impedance spectrum of HP-ZIF67-G/PANI/CC and HP-ZIF67-G/CC.

To further explore the interaction between polyaniline and HP-ZIF-67-G, we also tested the properties of pure polyaniline and HP-ZIF-67-G under the same conditions. The results indicate that the capacitance of the PANI/CC device is only 625 F cm^−2^ (Figure S11b) and that of the HP-ZIF-67-G/CC device is 15 mF cm^−2^ (Figure S9d). Based on the above, it can be concluded that the performance of the HP-ZIF67-G/PANI/CC device is higher than the sum of pure PANI and HP-ZIF67-G. This indicates that there exists a significant synergistic effect between ZIF-67 and PANI. The superior performance of the device can be attributed to the inherent porous properties of HP-ZIF67-G and the presence of a PANI electron transport bridge between the external circuit and the inner surface of the MOF. This enhances the electrical conductivity of the hybrid electrode. This is further supported by the impedance data presented in Figure 9. The Nyquist plots display a semicircle in the high-frequency region and a straight line in the low-frequency region. From the simulation, it is evident that the series resistance (R_s_) of HP-ZIF67-G/PANI/CC increases from 0.79 Ω to 1.74 Ω, but the charge transfer resistance (R_ct_) significantly decreases from 32.9 Ω to 5.743 Ω as compared to HP-ZIF67-G/CC. These results demonstrate that the incorporation of polyaniline greatly enhances the conductivity of the MOF material, thereby improving the overall performance of the supercapacitor. Thus, supercapacitor devices with excellent performance were obtained.

## 4. Conclusions

A novel gelation approach to the large-scale synthesis of MOF particles with a hierarchical porous structure was reported for the first time. The prepared ZIF-67 gel precursor has a good mechanical response and ductility. The synthesized HP-ZIF67-G has a high specific surface area of 1765 m^2^ g^−1^, and the supercapacitors based on HP-ZIF67-G/PANI hybrid electrodes show high specific surface capacitances in both solid and liquid electrolytes, which are above the value of 2500 mF cm^−2^. The synergistic interaction between ZIF-67 and PANI lead to the superior performance of the device, with PANI chains acting as a bridge for electron transport between the external circuit and the inner surface of the MOF particles. These results demonstrate that HP-ZIF67-G gel is a promising electrode material for energy storage devices. This work should also benefit the design of new hierarchical porous MOFs and stimulate further applications in a wide spectrum of fields of energy storage, catalysis or chemical sensors.

## Data Availability

Data available on request from the authors.

## Supplementary Materials

The following supporting information can be downloaded at: https://www.mdpi.com/article/10.3390/nano13101691/s1, Table S1 and Figure S1: The influence of raw material concentration and proportion on product shape; Figure S2: SEM images of ZIF-67 crystals; Figure S3: TEM of ZIF-67 growth without trimethylamine; Figure S4: The digital images of HP-ZIF67-G/PANI/CC. Figure S5: CV curve of polyaniline electro-deposition; Figure S6: EDS of HP-ZIF67-G/PANI/CC; Figure S7: The digital images of symmetric supercapacitor devices; Figure S8: CV curve of capacitance contribution; Figure S9: capacitor performance of HP-ZIF67-G/CC and ZIF-67/CC in solid electrolyte; Figure S10: capacitor performance of HP-ZIF67-G/PANI/CC in liquid electrolyte; Figure S11: capacitor performance of PANI/CC in gel electrolyte.
