# Supplementary material for: Large-Scale Synthesis of Hierarchical Porous MOF Particles via a Gelation Process for High Areal Capacitance Supercapacitors
Source: Nanomaterials (Basel). 2023 May 21;13(10):1691. doi: 10.3390/nano13101691 (PMC10222777; doi:10.3390/nano13101691)
Supplement: Supplementary file 1 [file nanomaterials-13-01691-s001.zip › nanomaterials-2388473-supplementary.pdf]

Supporting Information

Large-Scale Synthesis of Hierarchical Porous MOF Particles via a Gelation Process for High Areal Capacitance Supercapacitors

Yujie Sun [1], Fei Shi [1], Bo Wang [1], Naien Shi [1,2,*], Zhen Ding [1], Linghai Xie [1], Jiadong Jiang [2] and Min Han [2,*]

[1] State Key Laboratory of Organic Electronics and Information Displays, Institute of Advanced Materials, Nanjing University of Posts & Telecommunications, Nanjing 210023, China; 13523799407@139.com (Y.S.); 18151039127@163.com (F.S.); bowangxiao@163.com (B.W.); 2019060112@njupt.edu.cn (Z.D.); iamlhxie@njupt.edu.cn (L.X.)

[2] Strait Laboratory of Flexible Electronics (SLoFE), Strait Institute of Flexible Electronics (SIFE, Future Technologies), Fujian Normal University, Fuzhou 350117, China; jiangjiadong12345@gmail.com

* Correspondence: iamneshi@njupt.edu.cn (N.S.); ifemhan@fjnu.edu.cn (M.H.)

Table S1. The influences of the concentration and proportion of raw materials.

No.	$Co(NO_3)_2$ (mmol)	2-MIM (mmol)	TMA (30 wt%, mL)	ethanol (mL)	water (mL)	character
①	1.8	7.2	1.1	2.4	0	Gel
②	1.8	3.6	1.1	2.4	0	Gel-like
③	0.9	7.2	1.1	2.4	0	Sol
④	0.9	3.6	1.1	2.4	0	Sol
⑤	2.4	9.6	1.1	2.4	0	Gel+Sol
⑥	2.4	9.6	1.1	0	2.4	Sol

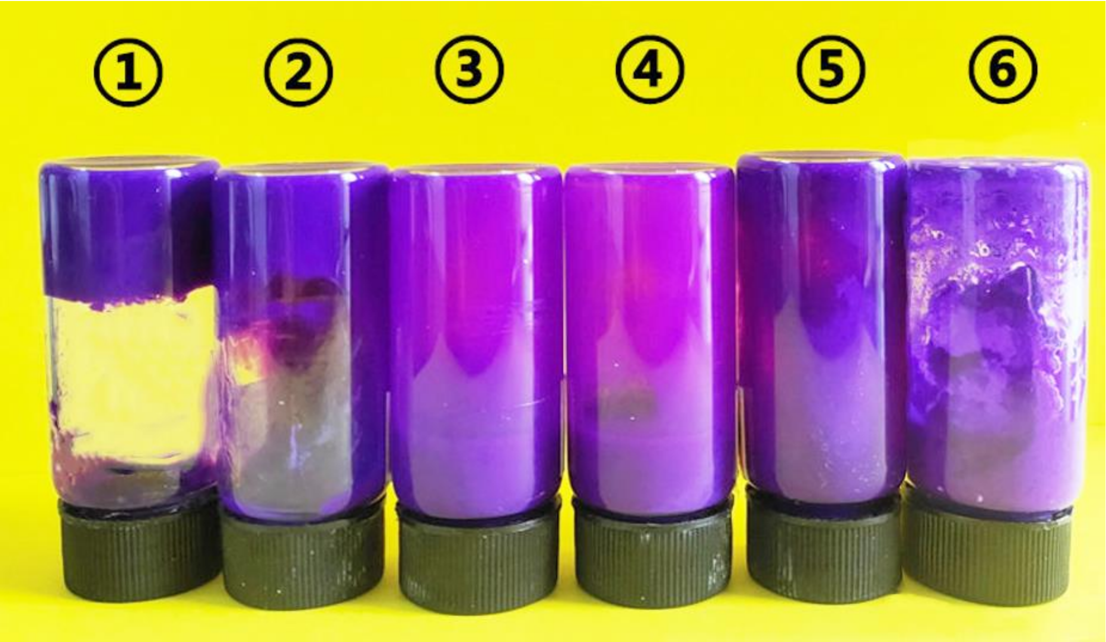

Figure S1. Photographs of the obtained products shown in Table S1.

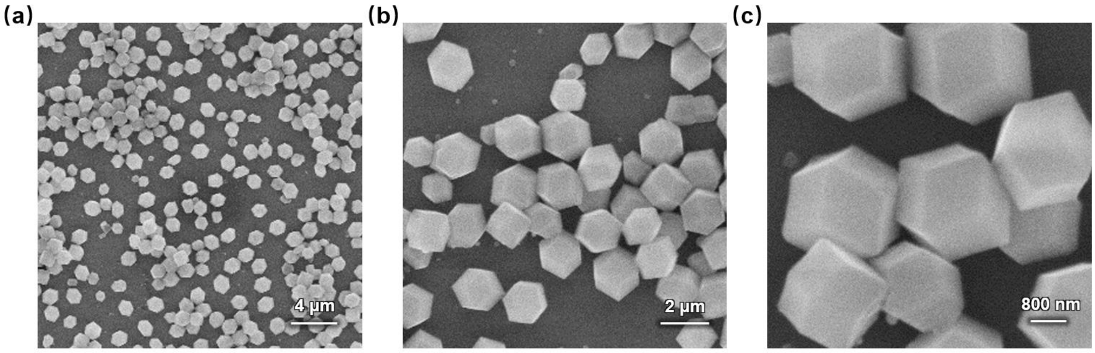

Figure S2. (a-c) SEM images of ZIF-67 crystals.

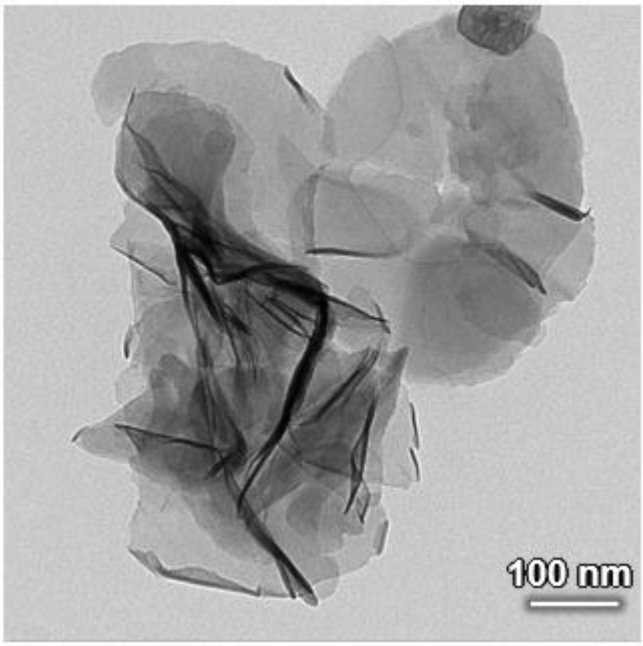

Figure S3 TEM image of ZIF-67 growth without trimethylamine.

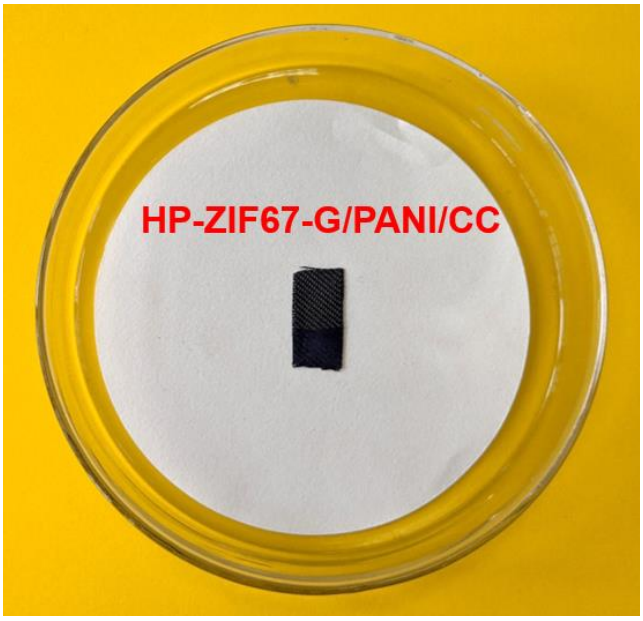

Figure S4. Photograph of HP-ZIF67-G/PANI/CC.

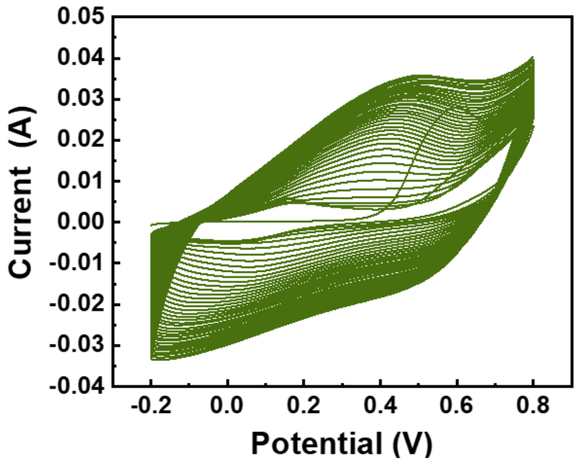

Figure S5. CV curves during PANI electro-deposition.

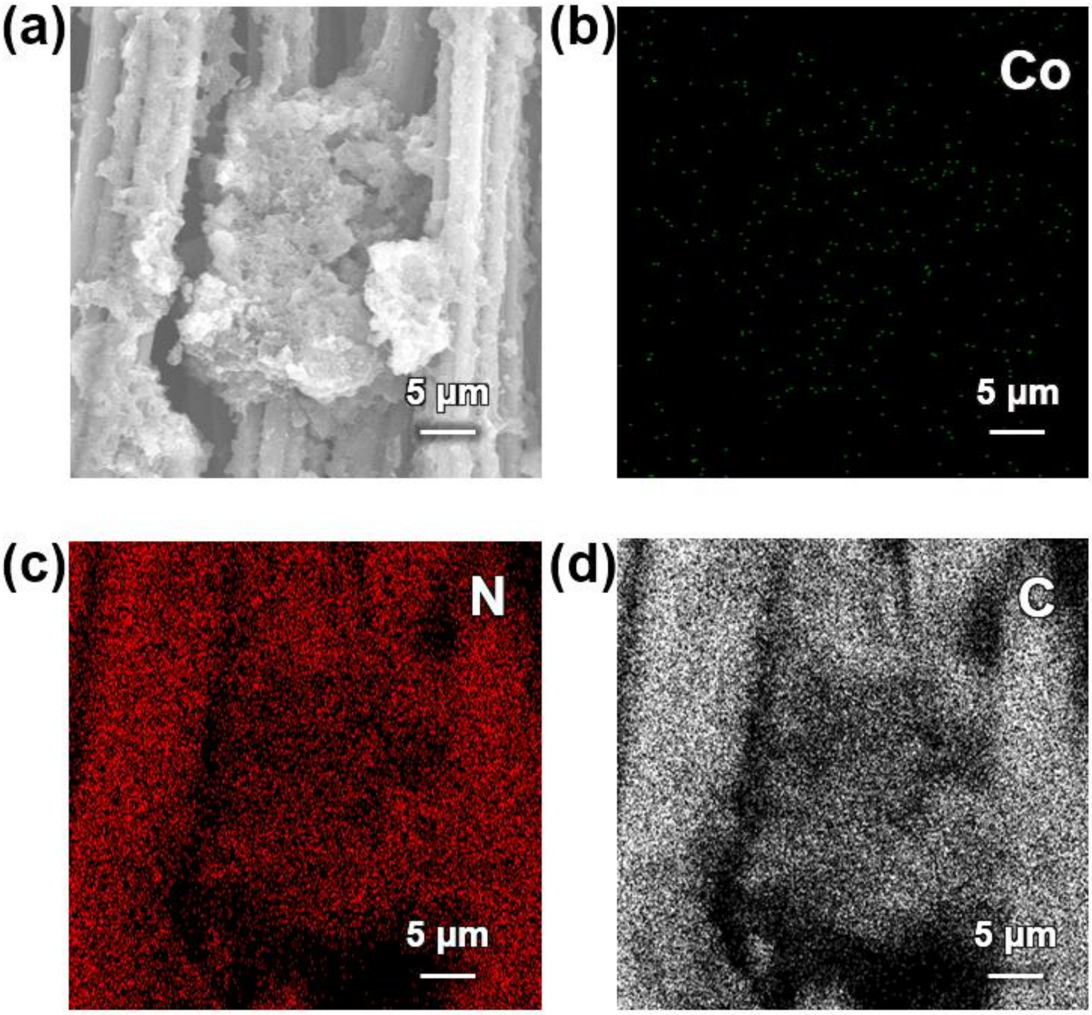

Figure S6. Elemental mapping images of HP-ZIF67-G/PANI/CC.

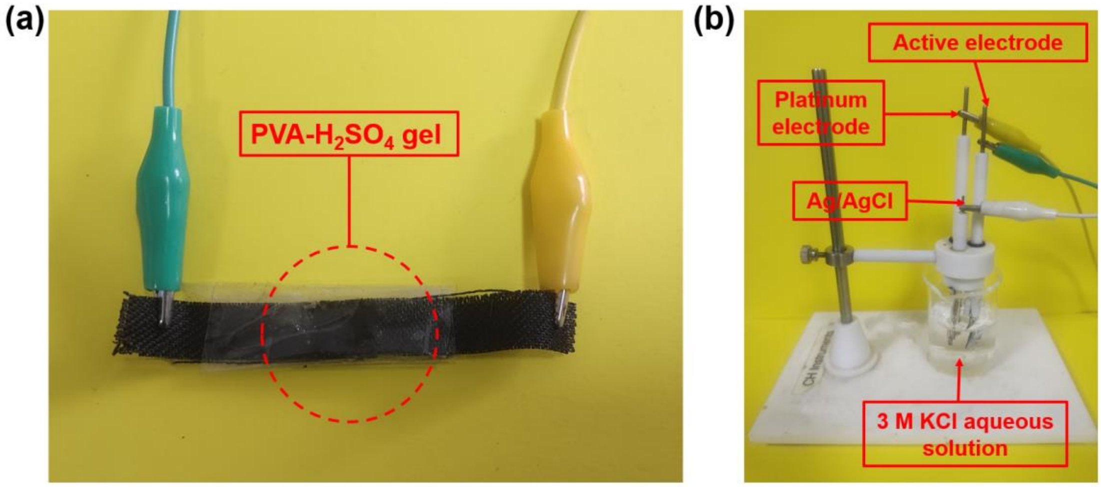

Figure S7. (a) Photograph of the symmetric supercapacitor device; (b) Photograph of the liquid supercapacitor device.

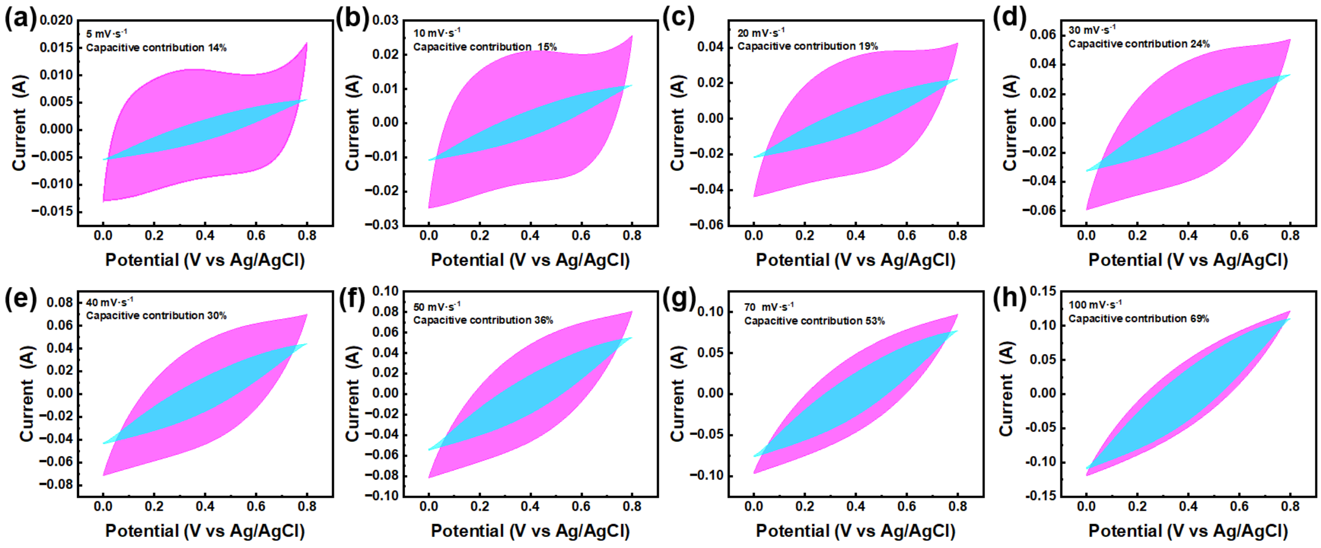

Figure S8. Capacitive contribution of ZIF67/PANI/CC electrode in KCl aqueous solution electrolyte at various scan rates.

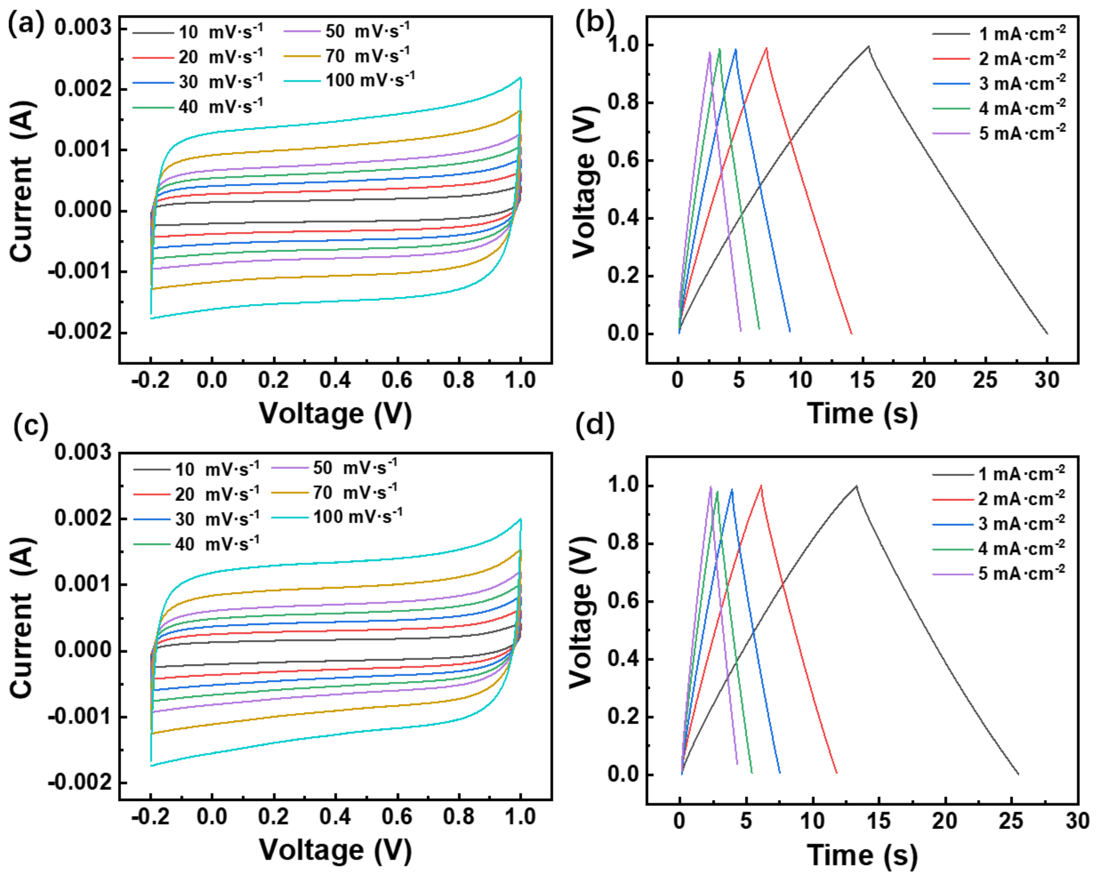

Figure S9. (a) CV curves of HP-ZIF67-G/CC electrode in PVA-H_2SO_4 gel electrolyte; (b) GCD curves of HP-ZIF67-G/CC in PVA-H_2SO_4 gel electrolyte; (c) CV curves of ZIF-67/CC in PVA-H_2SO_4 gel electrolyte; (d) GCD curves of ZIF-67/CC in PVA-H_2SO_4 gel electrolyte.

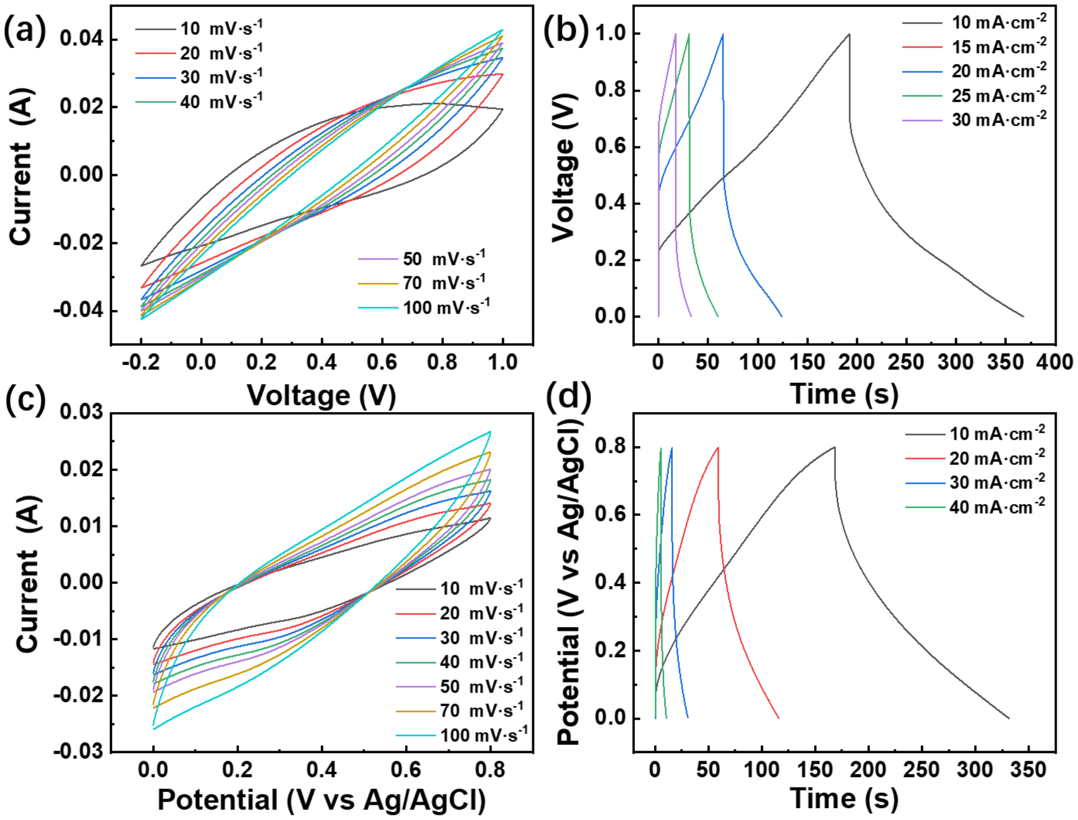

Figure S10. (a) CV curves of ZIF-67/PANI/CC electrode in PVA-H_2SO_4 gel electrolyte; (b) GCD curves of ZIF-67/PANI/CC in PVA-H_2SO_4 gel electrolyte. (c) CV curves of ZIF-67/PANI/CC electrode in KCl aqueous solution electrolyte; (d) GCD curves of ZIF-67/PANI/CC in KCl aqueous solution electrolyte.

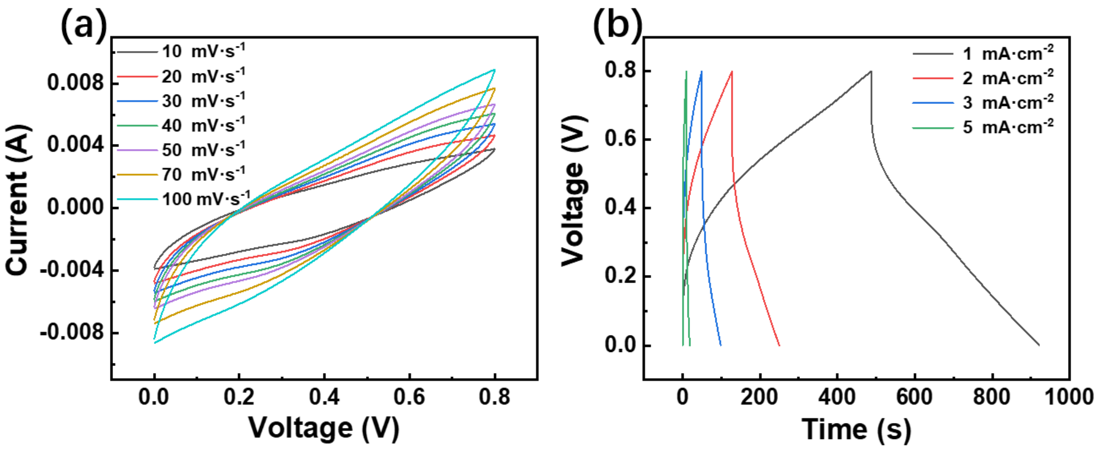

Figure S11. (a) CV curves of PANI/CC electrode in PVA-H_2SO_4 gel electrolyte; (b) GCD curves of PANI/CC electrode in PVA-H_2SO_4 gel electrolyte.
